# Clinical Application of Vascular Regenerative Therapy for Peripheral Artery Disease

**DOI:** 10.1155/2013/179730

**Published:** 2013-11-24

**Authors:** Hiroshi Suzuki, Yoshitaka Iso

**Affiliations:** ^1^Division of Cardiology, Department of Internal Medicine, Showa University, Fujigaoka Hospital, 1–30 Fujigaoka, Aoba-ku, Yokohama Kanagawa 227-8501, Japan; ^2^Department of Internal Medicine, Showa University Fujigaoka Rehabilitation Hospital, 2-1-1 Fujigaoka, Aoba-ku, Yokohama Kanagawa 227-8501, Japan

## Abstract

Prognosis of peripheral artery disease (PAD), especially critical limb ischemia, is very poor despite the development of endovascular therapy and bypass surgery. Many patients result in leg amputation and, therefore, vascular regenerative therapy is expected in this field. Gene therapy using vascular endothelial growth factor is the first step of vascular regenerative therapy, but did not confirm effectiveness in a large-scale randomized comparative study. Based on animal experiments, bone marrow mononuclear cells (MNCs), peripheral blood MNCs were used as the cell source for regenerative therapy. Those cells were confirmed to be effective to decrease rest pain and ulcer size, but its effect was not fully satisfied. Mesenchymal stem cells (MSCs) are expected as an effective cell source for vascular regeneration and clinical studies are ongoing, because the cells are able to differentiate into various cell types and produce a significant amount of vascular growth factors. Of vascular regeneration therapy, peripheral MNCs and bone marrow MNCs were recognized as advanced medical technology but do not attain to the standard therapy. However, clinical use of MSCs have already started, and induced pluripotent stem cells are surely promising tool for vascular regeneration therapy although further basic studies are required for clinical application.

## 1. Introduction

More than 25 million people have peripheral arterial disease (PAD) in Europe and the United States [1–5] and it is difficult to accurately understand the number of amputees. The incidence has been increasing also in Japan and the percentage of Buerger's disease was high before; however, Buerger's disease is currently rare and arteriosclerosis obliterans (ASO) is predominant, suggesting changes in disease structure. In treatment of PAD, control of coronary risk factors such as hypertension, diabetes, dyslipidemia, and smoking cessation is performed changing life style and prescribing medicine, and pharmacotherapy using antiplatelet agents and vasodilators and endovascular therapy using catheter intervention and bypass surgery are conducted [6–8]. Approximately 1% of patients with PAD result in critical limb ischemia (CLI) with serious prognosis. CLI is defined as a Rutherford Class 4–6 which is a condition characterized by rest pain, ulcers, and gangrene [9, 10]. Leg amputation is in more than 30% of patients with CLI for one year and the mortality is 20% or more [10]. Cardiovascular medicine has recently been developing and endovascular therapy with catheter intervention for PAD, particularly CLI, has been conducted for several years, and consequently, improved amputation-free survival was confirmed [11]. Nevertheless, leg amputation or death occurred in many patients and, therefore, vascular regenerative therapy is proactively conducted and expected in this field.

**Figure 1:**
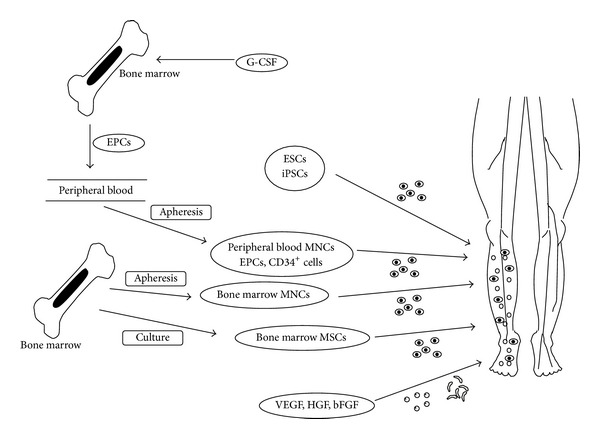
Angiogenic growth factors and cells for vascular regeneration. VEGF: vascular regenerative therapy, HGF: hepatocyte growth factor, bFGF: basic fibroblast growth factor, MNCs: mononuclear cells, EPCs: endothelial progenitor cells, MSCs: mesenchymal stem cells, ESCs: embryonic stem cells, iPSCs: induced pluripotent stem cells, and G-CSF: granulocyte colony stimulating factor.

For cardiovascular regenerative therapy, gene therapy for PAD using vascular endothelial growth factor (VEGF) started in 1996 in the United States [12]. The efficacy of gene therapy was expected in a small-scale clinical study but not confirmed in a large-scale randomized comparative study. Nevertheless, gene therapy made a great step towards cardiovascular regenerative therapy. Subsequently, the efficacy of bone marrow mononuclear cells (MNCs) [13] and endothelial progenitor cells (EPCs) [14] for vascularization was confirmed in several animal studies. The efficacy of MSCs was confirmed also in the first clinical study [15], but its effect is not fully sufficient in large scale study conducted in Japan [16], and the other cells for vascular regeneration are expected. In somatic stem cells, mesenchymal stem cells (MSCs) which are able to differentiate into various cell types and produce a significant amount of cytokines and vascular growth factors as compared to MNCs or EPCs are expected as an effective cell and clinical studies are ongoing. Human embryonic stem cells (ESCs) were produced also in Japan and many basic studies were conducted; however, clinical application was restricted due to bioethical concerns. However, induced pluripotent stem cells (iPSCs) were discovered and are expected to apply to clinical practice because bioethical concerns and rejection are resolved (Figure 1).

## 2. Angiogenic Growth Factors and Cells for Vascular Regeneration

### 2.1. Gene Therapy

Gene therapy of cancer is about 65% of those performed in the world; on the other hand, that of cardiovascular disease is about 9%, second-ranked. Adenovirus and retrovirus are major viral vectors; however, accidental death occurred in the United States after intracoronary delivery of adenovirus in 1999 [17] and leukemia occurred in France due to gene therapy in 2003 [18]; consequently, the safety is not established yet. Human recombinant protein was administered in various manners without gene vector such as intra-arterially and intravenously; consequently, ischemic improvement including increased collateral circulation was confirmed in animal models. However, an enormous amount of protein is necessary for these effects and this treatment is unrealistic for clinical use. On the other hand, naked/plasmid DNA and lipofectin were considered not to be superior to viral vector due to low transfection efficiency; however, Isner et al. showed the effect of VEGF on ischemic improvement using easy-to-use treatment with intramuscular injection of plasmid without viral vector [19] and confirmed the effect of plasmid on patients with PAD for the first time in 1996 [12, 20]. However, in a large-scale study, some of subjects showed that insufficient efficacy and vascular hyperpermeability induced by VEGF caused adverse reaction, edema. Consequently, this is not recognized as standard therapy in practice.

Hepatocyte growth factor (HGF) is known to regulate proliferation and migration of vascular endothelial cells via c-met, its receptor. HGF was confirmed to have angiogenic activity similarly to VEGF and a small size clinical study for patients with PAD confirmed the efficacy and safety of HGF [21]. Also in a randomized, double-blind, clinical trial in patients with serious PAD of Fontaine stage III/IV who could not undergo revascularization with HGF plasmid, rest pain and ulcer size significantly decreased in the HGF group in comparison with those in the control group [22]. 

Basic fibroblast growth factor (bFGF or FGF-2) is attracting attention similarly to VEGF and HGF or more. An animal study showed that local VEGF concentration after administration increased up to severalfold and caused adverse reactions including edema and hemorrhage without effective angiogenic activity; in contrast, FGF-2 caused no adverse reaction at high doses and had efficacy already at low doses. In histological examination, angiogenesis caused by vascular endothelial cell marker was similar between the two groups; however, significantly more *α*-smooth muscle actin-positive vessels were observed in the FGF-2 group significantly more frequently and it was confirmed that mature and functional angiogenesis was established [23]. On the other hand, this effect of FGF-2 was also evidenced to be involved in strong endogenous inducing action of VEGF-A and HGF [23, 24]. A study using Sendai virus as a vector of FGF-2 is ongoing because Sendai virus is not transformed into DNA and transcription and genome replication are conducted in cytoplasm; therefore, it expresses genes without influence to chromosome of host cells. Phase I/IIa open-label clinical study using recombinant Sendai virus vector with human FGF-2 gene (rSeV/dF-hFGF-2) was completed and showed its safety, improved Rutherford category and pain scale, and extended walk distance. A larger-scale study is planned [25].

### 2.2. CD34^+^ Cells and Endothelial Progenitor Cells (EPCs)

Angiogenesis in adults was considered to be produced by proliferation and migration of adjacent existing microvascular endothelial cells. However, in 1997, it was found that EPCs differentiating into endothelial cells existed in monocytes of adult peripheral blood [26] and vasculogenesis in ischemic sites caused by bone marrow-derived EPCs was confirmed to be implicated in angiogenesis in adults. In a study in which EPCs derived from cultured peripheral blood mononuclear cells of healthy volunteers were intravenously administered to limb ischemia nude mouse model, grafted cells accumulated in ischemic sites and histology confirmed increased capillary density and laser-Doppler flowmetry evidenced improved lower limb blood flow [14]. Similar effects were shown also in grafting of human cord blood-derived EPC nude mouse model with EPCs limb ischemia [27]. Granulocyte colony stimulating factor (G-CSF) was subcutaneously given to patients with CLI of Fontaine stage III/IV who could not undergo revascularization and then monocytes that were mobilized from bone marrow to peripheral blood were collected using apheresis and CD34^+^ cells in monocytes were separated as EPC fraction and administered locally to the muscle of CLI [28]. Long-term safety and effect particularly on Buerger's disease have recently been shown [29].

### 2.3. Bone Marrow Mononuclear Cells (MNCs)

Bone marrow cells were confirmed to contain a plenty of EPCs and secrete abundant angiogenic factors VEGF and bFGF. Consequently, autologous bone marrow transplantation to limb skeletal muscles in limb ischemia model enhanced angiogenesis and significantly increased lower limb blood flow [13]. Based on these results, a pilot study of intramuscular grafting of autologous bone marrow MNCs in patients with ischemic limb was conducted in Japan [15] and showed significant improvement in ankle brachial index and transcutaneous oxygen pressure by autologous bone marrow MNCs and significant prolongation of walking time in a treadmill exercise test for 6-month follow-up period. In a multicenter study that we participated in [16], intramuscular grafting in patients with CLI improved visual analogue scale, decreased ulcer size, and increased walk distance in both PAD and Buerger's disease groups and particularly visual analogue scale was confirmed to continue until at least 2 years after. This treatment was already approved as advanced medical technology by the Ministry of Health, Labour, and Welfare (MHLW). However, recurrence of ulcer was subsequently confirmed and walk distance decreased in some subjects of the PAD group; consequently, long-term effect is unclear. A multicenter, double-blind, randomized-start phase II trial using bone marrow MNCs (PROVASA) [30] assigned 40 patients with CLI to the bone marrow MNC and control groups and MNC was intra-arterially given into the femoral artery of affected side. ABI and amputation-free survival of the primary endpoints were similar in the two groups; however, ulcer size and pain scale significantly decreased within 3 months in the MNC group although no change occured in the control group.

### 2.4. Peripheral Blood Mononuclear Cells

Bone marrow MNCs have already been confirmed to be effective; however, the major issue of concern was the size of invasion to patients complicated with other cardiovascular disease who were frequently found in patients with CLI among patients with PAD. Consequently, MNCs were collected from peripheral blood without collecting bone marrow, administered to the ischemic limb, and confirmed to be effective [31]. An angiogenic factor secreted from administered MNCs is probably a key for its efficacy; however, cytokines secreted from administered MNCs were confirmed to be insufficient for angiogenesis and stimulated muscular cells produce IL-1*β*, which was the main cause of angiogenesis [31]. Peripheral blood MNC grafting has already been used as clinical application and is currently approved as advanced by MHLW. Long-term outcomes of peripheral blood MNC grafting for CLI were shown in a study other than placebo control study that pain scale, walk distance, and ulcer size were significantly improved and the grafting was more effective and safe than conventional standard therapies [32]. In this study, ischemic symptoms of dialysis patients with PAD were improved less than those of nondialysis patients and patients with Buerger's disease. Death, major amputation, and cardiovascular events were frequently found in dialysis patients with PAD.

In another study, collected peripheral blood mononuclear cells after intravenous administration of G-CSF inducing CD34^+^ cells into peripheral blood were injected into ischemic sites, and ischemic improvement was confirmed by thermography and plethysmography [33]. In a pooled analysis to compare long-term prognosis between bone marrow MNC graft and G-CSF mobilized peripheral blood MNC in patients with CLI, overall survival and amputation-free survival were similar between the two groups and similar efficacy was confirmed. On the other hand, small amounts of CD34^+^ cells, hemodialysis, Fontaine classification, male, and age were factors for overall survival and amputation-free survival [34]. For long-term prognosis of G-CSF mobilized peripheral MNC, ischemic heart disease and small amounts of CD34^+^ cell were factors for overall survival [35].

### 2.5. Bone Marrow Mesenchymal Stem Cells (MSCs)

**Figure 2:**
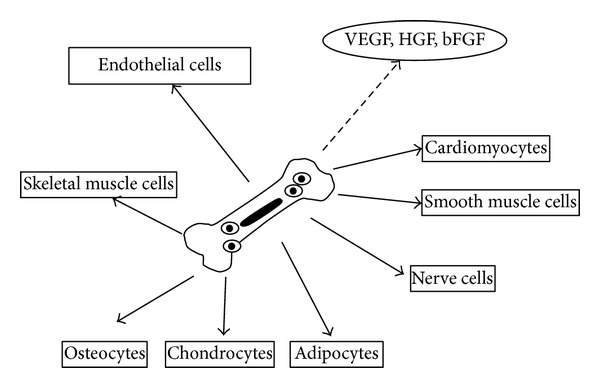
Differentiation capacity and production of angiogenic growth factors from bone marrow mesenchymal stem cells (MSCs). VEGF: vascular regenerative therapy, HGF: hepatocyte growth factor, and bFGF: basic fibroblast growth factor.

MSCs are included in not only bone marrow but also placenta, amnion, umbilical cord, and chorion and have been confirmed in vitro to differentiate into not only bone and cartilage but also myocardium and endothelial cells and inhibit cell death (Figure 2). Furthermore, MSCs can be cultured from a small amount of tissues and are attracting attention as a cellular source for cardiovascular revascularization [36–38]. In an experiment that bone marrow MNCs or MSCs were locally administered to limb ischemia rat model [39], local blood flow measured by laser-Doppler flowmetry 3 weeks after was larger in the MSC group and capillary density was significantly higher in the MSC group than that in the MNC group. The number of administered MSC-derived endothelial cells was significantly more than MNC-derived ones and administered cell-derived smooth muscle cells were observed only in the MSC group. The ratio of cell apoptosis under hypoxic conditions was significantly lower in the MSC group than that in the MNC group in an *in vitro* study and the MSC group was considered to be resistant to ischemia more than the MNC group. In a randomized, double-blind, controlled trial in patients with diabetes and CLI [40], pain-free walking time, ulcer size, and percutaneous tissue oxygen pressure were significantly improved in the MSC group in comparison with those in the MNC groups and production of VEGF and FGF-2 was significantly higher in the MSC group with conditioned medium than those in the MNC group. On the other hand, Iso et al. [36] conducted an animal study and confirmed that human MSC produced large amounts of growth factors including VEGF and adrenomedullin and cytokines and enhanced angiogenesis in comparison with animal MSC. VEGF and massive HGF were produced in the conditioned medium of rat bone marrow MSCs [41] and the major cause of ischemic improvement of MSCs was suggested to be actions of angiogenic factors such as VEGF and HGF. MSCs are known to be less antigenic due to defect of MHC class II and may be used for allogeneic implantation [41]. Rat fetal membrane-derived MSCs expressed cell surface antigens similar to bone marrow MSCs and VEGF production in the conditioned medium was low but HGF production was similar. Fetal membrane-derived MSCs were administered to limb ischemia rat model and had ischemic improvement similar to bone marrow MSCs and had local lymphocytic infiltration similar to bone marrow MSCs. Therefore, allogeneic implantation may be conducted any time by banking MSCs without functional problems that were derived from the placenta, amnion, umbilical cord, and chorion, not MSCs derived from elderly patients with PAD.

### 2.6. Embryonic Stem Cells (ESCs) and Induced Pluripotent Stem Cells (iPSCs)

ESCs are cell line produced from the inner cell mass of the early embryo and have high proliferative capacity differentiate to all types of cells. It was confirmed that grafting of ES cell-derived EPCs in the body enhanced angiogenesis [42]. However, ESCs are produced by disrupting fertilized eggs; therefore, ethical and legal concerns are discussed and immunologic rejection may occur in cell transplantation due to absence of self-derived cells. In addition, teratoma may occur due to contamination of undifferentiated cells. In 2006, iPSCs were produced from dermal fibroblasts [43] and iPS-derived Flk-1^+^ cells differentiate into endothelial cells and mural cells* in vitro* and were implemented in angiogenesis [44]. In a study in which mouse iPS-derived Flk-1^+^ cells were transplanted to limb ischemia nude mouse model, blood flow in the chronic phase increased in comparison with that of the control group and Flk-1^+^ cells were observed directly in ischemic tissues. In addition, VEGF mRNA expression was increased. Consequently, it was suggested that administered cells were implemented in vasculogenesis in addition to paracrine effects of increased cytokine production [45]. Studies for clinical application are expected; however, no clinical studies start due to risk of teratoma, difficulties in massive culture, and vessel-selective separation procedures.

## 3. Development of Transplantation Procedures 

Not only cells to administer but also development of transplantation procedures is very important issues to furthermore improve therapeutic efficacy. In conventional cell administration method, local administration, transfection efficiency, and surviving rate of grafted cells in ischemic area are low; consequently, cells are disappeared within several days. Cell viability should be improved to release more cytokines secreted from transplanted cells as long as possible. Extremely low differentiation rate of transplanted cells to endothelial cells and vessels is also important issue to improve.

## 4. Conclusions

Studies of vascular regeneration therapy started around 1990 and studies of myocardial regeneration have been also conducted. Of vascular regeneration therapy, some including peripheral MNCs and bone marrow MNCs were approved as advanced medical technology but do not attain to the standard therapy.

However, MSCs that are contained in many organs including bone marrow attract massive attention as a new type regenerative medicine including future banking due to potent cytokine production capacity and low antigenicity. On the other hand, iPSCs are surely promising tool for vascular regeneration therapy although far from clinical application; therefore, further basic studies are required for clinical application.

## References

[1] Criqui MH, Fronek A, Barrett-Connor E (1985). The prevalence of peripheral arterial disease in a defined population. *Circulation*.

[2] Fowkes FGR, Housley E, Cawood EHH, Macintyre CCA, Ruckley CV, Prescott RJ (1991). Edinburgh Artery Study: prevalence of asymptomatic and symptomatic peripheral arterial disease in the general population. *International Journal of Epidemiology*.

[3] McGrae McDermott M, Kerwin DR, Liu K (2001). Prevalence and significance of unrecognized lower extremity peripheral arterial disease in general medicine practice. *Journal of General Internal Medicine*.

[4] Belch JJF, Topol EJ, Agnelli G (2003). Critical issues in peripheral arterial disease detection and management: a call to action. *Archives of Internal Medicine*.

[5] Norgren L, Hiatt WR, Dormandy JA, Nehler MR, Harris KA, Fowkes FGR (2007). Inter-society consensus for the management of peripheral arterial disease (TASC II). *Journal of Vascular Surgery*.

[6] Hirsch AT, Haskal ZJ, Hertzer NR (2006). ACC/AHA guidelines for the management of patients with peripheral arterial disease (lower extremity, renal, mesenteric, and abdominal aortic): a collaborative report from the American Associations for Vascular Surgery/Society for Vascular Surgery, Society for Cardiovascular Angiography and Interventions, Society for Vascular Medicine and Biology, Society of Interventional Radiology. *Journal of Vascular and Interventional Radiology*.

[7] Weitz JI, Byrne J, Patrick Clagett G (1996). Diagnosis and treatment of chronic arterial insufficiency of the lower extremities: a critical review. *Circulation*.

[8] Rowlands TE, Donnelly R (2007). Medical therapy for intermittent claudication. *European Journal of Vascular and Endovascular Surgery*.

[9] Becker GJ, Palmaz JC, Rees CR (1990). Angioplasty-induced dissections in human iliac arteries: management with palmaz balloon-expandable intraluminal stents. *Radiology*.

[10] Norgren L, Hiatt WR, Dormandy JA, Nehler MR, Harris KA, Fowkes FGR (2007). Inter-society consensus for the management of peripheral arterial disease (TASC II). *European Journal of Vascular and Endovascular Surgery*.

[11] Iida O, Nakamura M, Yamauchi Y (2013). Endovascular treatment for infrainguinal vessels in patients with critical limb ischemia : OLIVE Registry, a Prospective, Multicenter Study in Japan with 12-month follow-up. *Circulation Cardiovascular Intervention*.

[12] Isner JM, Pieczek A, Schainfeld R (1996). Clinical evidence of angiogenesis after arterial gene transfer of phVEGF165 in patient with ischaemic limb. *The Lancet*.

[13] Iba O, Matsubara H, Nozawa Y (2002). Angiogenesis by implantation of peripheral blood mononuclear cells and platelets into ischemic limbs. *Circulation*.

[14] Kalka C, Masuda H, Takahashi T (2000). Transplantation of ex vivo expanded endothelial progenitor cells for therapeutic neovascularization. *Proceedings of the National Academy of Sciences of the United States of America*.

[15] Tateishi-Yuyama E, Matsubara H, Murohara T (2002). Therapeutic angiogenesis for patients with limb ischaemia by autologous transplantation of bone-marrow cells: a pilot study and a randomised controlled trial. *The Lancet*.

[16] Matoba S, Tatsumi T, Murohara T (2008). Long-term clinical outcome after intramuscular implantation of bone marrow mononuclear cells (therapeutic angiogenesis by cell transplantation [TACT] trial) in patients with chronic limb ischemia. *American Heart Journal*.

[17] Marshall E (1999). Gene therapy death prompts review of adenovirus vector. *Science*.

[18] Gansbacher B (2003). Report of a second serious adverse event in a clinical trial of gene therapy for X-linked severe combined immune deficiency (X-SCID). Position of the European Society of gene therapy (ESGT). *The Journal of Gene Medicine*.

[19] Takeshita S, Zheng LP, Brogi E (1994). Therapeutic angiogenesis. A single intraarterial bolus of vascular endothelial growth factor augments revascularization in a rabbit ischemic hind limb model. *Journal of Clinical Investigation*.

[20] Baumgartner I, Pieczek A, Manor O (1998). Constitutive expression of phVEGF165 after intramuscular gene transfer promotes collateral vessel development in patients with critical limb ischemia. *Circulation*.

[21] Morishita R, Aoki M, Hashiya N (2004). Safety evaluation of clinical gene therapy using hepatocyte growth factor to treat peripheral arterial disease. *Hypertension*.

[22] Shigematsu H, Yasuda K, Iwai T (2010). Randomized, double-blind, placebo-controlled clinical trial of hepatocyte growth factor plasmid for critical limb ischemia. *Gene Therapy*.

[23] Masaki I, Yonemitsu Y, Yamashita A (2002). Angiogenic gene therapy for experimental critical limb ischemia: acceleration of limb loss by overexpression of vascular endothelial growth factor 165 but not of fibroblast growth factor-2. *Circulation Research*.

[24] Onimaru M, Yonemitsu Y, Tanii M (2002). Fibroblast growth factor-2 gene transfer can stimulate hepatocyte growth factor expression irrespective of hypoxia-mediated downregulation in ischemic limbs. *Circulation Research*.

[25] Yonemitsu Y, Matsumoto T, Itoh H (2013). DVC1-0101 to treat peripheral arterial disease: a Phase I/IIa open-label dose-escalation clinical trial. *Molecular Therapy*.

[26] Asahara T, Murohara T, Sullivan A (1997). Isolation of putative progenitor endothelial cells for angiogenesis. *Science*.

[27] Murohara T, Ikeda H, Duan J (2000). Transplanted cord blood-derived endothelial precursor cells augment postnatal neovascularization. *Journal of Clinical Investigation*.

[28] Kawamoto A, Katayama M, Handa N (2009). Intramuscular transplantation of G-CSF-mobilized CD34+ cells in patients with critical limb ischemia: a phase I/IIa, multicenter, single-blinded, dose-escalation clinical trial. *Stem Cells*.

[29] Kinoshita M, Fujita Y, Katayama M (2012). Long-term clinical outcome after intramuscular transplantation of granulocyte colony stimulating factor-mobilized CD34 positive cells in patients with critical limb ischemia. *Atherosclerosis*.

[30] Walter DH, Krankenberg H, Balzer JO (2011). Intraarterial administration of bone marrow mononuclear cells in patients with critical limb ischemia a randomized-start, placebo-controlled pilot trial (PROVASA). *Circulation*.

[31] Tateno K, Minamino T, Toko H (2006). Critical roles of muscle-secreted angiogenic factors in therapeutic neovascularization. *Circulation Research*.

[32] Moriya J, Minamino T, Tateno K (2009). Long-term outcome of therapeutic neovascularization using peripheral blood mononuclear cells for limb ischemia. *Circulation*.

[33] Kawamura A, Horie T, Tsuda I (2006). Clinical study of therapeutic angiogenesis by autologous peripheral blood stem cell (PBSC) transplantation in 92 patients with critically ischemic limbs. *Journal of Artificial Organs*.

[34] Onodera R, Teramukai S, Tanaka S (2011). Bone marrow mononuclear cells versus G-CSF-mobilized peripheral blood mononuclear cells for treatment of lower limb ASO: pooled analysis for long-term prognosis. *Bone Marrow Transplantation*.

[35] Horie T, Onodera R, Akamastu M (2010). Long-term clinical outcomes for patients with lower limb ischemia implanted with G-CSF-mobilized autologous peripheral blood mononuclear cells. *Atherosclerosis*.

[36] Iso Y, Spees JL, Serrano C (2007). Multipotent human stromal cells improve cardiac function after myocardial infarction in mice without long-term engraftment. *Biochemical and Biophysical Research Communications*.

[37] Amado LC, Saliaris AP, Schuleri KH (2005). Cardiac repair with intramyocardial injection of allogeneic mesenchymal stem cells after myocardial infarction. *Proceedings of the National Academy of Sciences of the United States of America*.

[38] Lim SY, Kim YS, Ahn Y (2006). The effects of mesenchymal stem cells transduced with Akt in a porcine myocardial infarction model. *Cardiovascular Research*.

[39] Iwase T, Nagaya N, Fujii T (2005). Comparison of angiogenic potency between mesenchymal stem cells and mononuclear cells in a rat model of hindlimb ischemia. *Cardiovascular Research*.

[40] Lu D, Chen B, Liang Z (2011). Comparison of bone marrow mesenchymal stem cells with bone marrow-derived mononuclear cells for treatment of diabetic critical limb ischemia and foot ulcer: a double-blind, randomized, controlled trial. *Diabetes Research and Clinical Practice*.

[41] Ishikane S, Ohnishi S, Yamahara K (2008). Allogeneic injection of fetal membrane-derived mesenchymal stem cells induces therapeutic angiogenesis in a rat model of hind limb ischemia. *Stem Cells*.

[42] Yurugi-Kobayashi T, Itoh H, Yamashita J (2003). Effective contribution of transplanted vascular progenitor cells derived from embryonic stem cells to adult neovascularization in proper differentiation stage. *Blood*.

[43] Takahashi K, Yamanaka S (2006). Induction of pluripotent stem cells from mouse embryonic and adult fibroblast cultures by defined factors. *Cell*.

[44] Narazaki G, Uosaki H, Teranishi M (2008). Directed and systematic differentiation of cardiovascular cells from mouse induced pluripotent stem cells. *Circulation*.

[45] Suzuki H, Shibata R, Kito T (2010). Therapeutic angiogenesis by transplantation of induced pluripotent stem cell-derived Flk-1 positive cells. *BMC Cell Biology*.

